# Correction of stomach cancer CT attenuation values for variations due to differences in CT imaging conditions through repeated CT scans

**DOI:** 10.1371/journal.pone.0321085

**Published:** 2025-04-24

**Authors:** Jeong Min Seo, Sun Young Baek, Woo Kyoung Jeong, Kyoung Doo Song

**Affiliations:** 1 Department of Radiology, Samsung Medical Center, Sungkyunkwan University, Seoul, Korea; 2 Biomedical Statistics Center, Research Institute for Future Medicine, Samsung Medical Center, Seoul, Korea; Universiti Teknologi Malaysia - Main Campus Skudai: Universiti Teknologi Malaysia, MALAYSIA

## Abstract

**Purpose:**

To develop methods for correcting variations in CT attenuation values of advanced gastric cancer (AGC) due to differences in CT imaging conditions using repeated pre-treatment CT scans.

**Methods:**

A total 211 patients (146 men) with AGC who underwent pre-treatment CT twice were included in this retrospective study. The Pearson correlation between the difference in tumor attenuation values measured on both CT scans and the difference in attenuation values of other organs was analyzed. A formula to correct tumor CT attenuation values was developed using univariate linear regression analysis.

**Results:**

The Pearson correlation coefficient was the highest between the difference in tumor attenuation values and that of the main portal vein (MPV) attenuation values (0.86, *P* <.01). The formula to correct tumor attenuation values was as follows: calculated tumor attenuation value on CT scan 2 = tumor attenuation value on CT scan 1 - (-3.5 + 0.4 x (MPV attenuation value on CT scan 1 - MPV attenuation value on CT scan 2)). The mean difference between calculated and actual tumor attenuation values was 1.6 HU (SD, 8.7; range -22.5–24.72), with a Pearson correlation coefficient of 0.95 (*P* <.01).

**Conclusion:**

Utilizing the attenuation value of the MPV allows for correction of variations in tumor attenuation values caused by different CT imaging conditions, enabling the prediction of reproducible tumor attenuation in patients with AGC. Future studies are needed to validate these findings and address the study’s limitations, including its retrospective design and the absence of unenhanced CT data.

**Advances in knowledge:**

The attenuation value of the MPV can be used to predict reproducible tumor attenuation values in gastric cancer.

## Introduction

Gastric cancer is one of the most common cancers and the third leading cause of cancer-related deaths worldwide [1]. Computed tomography (CT) is the modality of choice in the management of gastric cancer, essential for diagnosis, staging, and response monitoring. The degree of contrast enhancement on CT reflects tumor characteristics such as the extent of fibrosis or angiogenesis, which are critical factors in tumor growth, invasion, and metastasis. This feature can be used to classify the tumor phenotype or evaluate the response to drugs such as anti-angiogenic agents [2,3]. For instance, Choi criteria use tumor attenuation as well as tumor size on CT to define the treatment response of gastrointestinal stromal tumors [4]. This criterion was also applied to patients with metastatic renal cell carcinoma treated with targeted therapies and was superior in discriminating prognostic groups based on tumor size alone [5].

However, the degree of contrast enhancement on CT is influenced by multiple factors, including the patient’s overall status, contrast agent (dose and infusion rate), and CT scanning parameters (imaging acquisition time and tube voltage) [6,7]. These factors can lead to significant inconsistencies in the quantitative assessment of tumor enhancement, despite controlled and repeated scanning conditions. For example, Dercle et al. [8] reported that the overall decrease in tumor attenuation attributable to nonoptimal portal venous phase timing was significant at 14.8%. Consequently, the lack of reproducibility in measuring the degree of contrast enhancement of tumors is an obstacle to its clinical use in monitoring disease progression or therapeutic response over time.

To address the variability in measuring the CT attenuation value of tumors, Dercle et al. investigated the relationship between the attenuation of vascular structures and the attenuation of liver metastases from colon cancer [8]. Their study demonstrated that portal vein attenuation is associated with the attenuation of liver metastases. However, the blood supply and drainage of the liver and stomach differ. Additionally, their study utilized only a single CT scan per patient, making it challenging to account for inter-patient differences and variations in CT imaging conditions. We hypothesized that by analyzing repeated CT scans taken at short intervals in the same patient, we could correct for variations in contrast enhancement due to differences in patient or CT imaging condition and predict contrast enhancement attributable to the intrinsic characteristics of the tumor.

This study aimed to develop methods for correcting variations in CT attenuation values of advanced gastric cancer (AGC) due to differences in CT imaging conditions using repeated pre-treatment CT scans.

## Materials and methods

This retrospective study was approved by the Institutional Review Board of Samsung Medical Center (SMC IRB 2024-02-105-001). The requirement for an informed consent was waived because the study involved a retrospective review of medical records and images. The data were accessed from March 1, 2024 to June 30, 3024 for research purposes. The first author and corresponding author had access to information that could identify individual participants during data collection.

### Patients

Between January 2014 and December 2023, 796 patients with gastric cancer underwent contrast-enhanced CT twice within a one-month interval before treatment: once at an outside institution and once at our institution. The reason for repeated CT gastrography at our institution was that the surgeons determined that a dedicated gastric cancer CT was necessary for surgical planning. Among them, 585 patients were excluded for the following reasons: gastric cancer lesions not visible on the CT scan (n = 552), lesion size too small for an accurate attenuation measurement (n = 30), poor image quality of outside CT scan (n = 2), and no portal venous phase on outside CT scan (n = 1). Finally, 211 patients (65 women and 146 men; mean age, 62 years ± 12) were included in this study (Fig 1).

**Fig 1:**
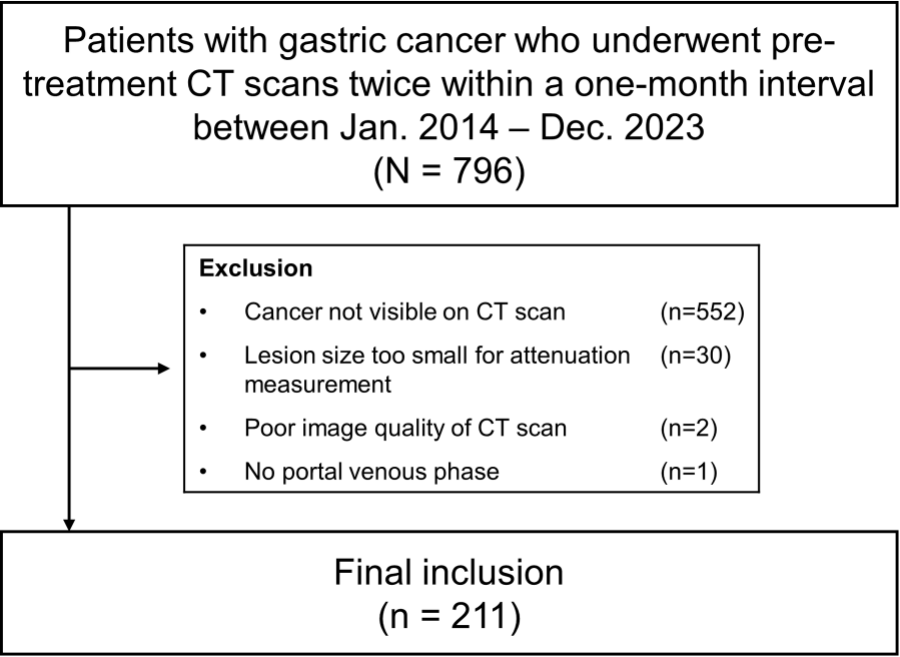
Inclusion flow chart.

### Image acquisition

CT gastrography was performed using one of the following multi-detector-row CT scanners: LightSpeed VCT, Discovery CT 750HD, Revolution Frontier, or Revolution Apex of GE Healthcare (Milwaukee, WI, USA). Before CT the examination, patients ingested effervescent granules 8 g with 10mL of tap water to obtain gastric distention. Arterial phase CT was performed with patients in the right lateral decubitus position, 40 seconds after the initiation of contrast material injection (typically 120 mL of 300 mg I/mL at 4 mL/s), and portal venous phase CT was performed with patients in the supine position 70 seconds after the initiation of contrast material injection. The scanning parameters were as follows: tube voltage, 120 kVp; tube current, 120–350 mA; and pitch factor, 0.984–1.375. Automated tube current modulation was used. The slice thickness was 3.5 mm for the axial images and 3 mm for the coronal and sagittal images. 3D CT gastrography was obtained using surface-shaded volume-rendering technique. CT scans at outside institutions were performed using various CT scanners and CT acquisition protocols: tube voltage ranged from 80 kVp to 140 kVp, and slice thickness ranged from 1 mm to 5 mm. Detailed image acquisition information for outside CT scans, such as the exact timing of the portal venous phase and the amount of contrast agent used, was not available.

### Measurement of CT attenuation value

One researcher (a medical illustrator with four years of experience in the segmentation of abdominal organs and lesions) segmented gastric cancer, aorta, main portal vein, inferior vena cava (IVC), spleen, and back muscle on a picture archiving and communication system (PACS; Centricity Radiology RA 1000; GE Healthcare, Chicago, IL, USA) to measure attenuation values, and an abdominal radiologist with 15 years of experience confirmed the segmentation results. Segmentation was performed manually using the built-in functionality of the PACS. While segmenting all slices containing the tumor may theoretically be preferable to account for tumor heterogeneity and variability in CT attenuation values due to slice selection, it is often challenging to clearly define the tumor boundaries in all slices for stomach cancer, and artifact can distort the CT attenuation values in some slices. For these reasons, the average CT attenuation value for the tumor was measured by segmenting the entire tumor on the slice containing the most tumor and had no artifacts, among axial, coronal, and sagittal images (Fig 2). The average CT attenuation value of the main portal vein was measured at the mid-portion of the main portal vein on axial images. The average CT attenuation values for the aorta, IVC, spleen, and back muscle were measured in the same slice where the attenuation of the main portal vein was measured. If the spleen was not covered in that slice, the average CT attenuation value of the spleen was measured by segmenting the entire spleen on the slice with the largest portion of the spleen. The average CT attenuation value of the back muscle was measured by drawing a region of interest of at least 200 mm^2^. To evaluate the interobserver agreement for measuring CT attenuation values, one radiologic resident measured the CT attenuation values in the same manner for 45 patients independently.

**Fig 2:**
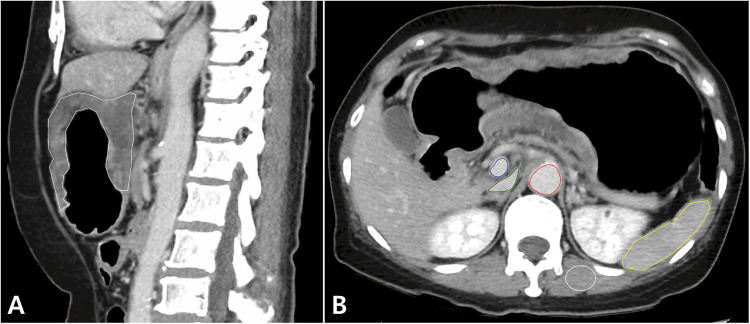
Representative case of measurement of CT attenuation. A. Measurement of CT attenuation value of gastric cancer, sagittal portal venous phase image. B. Measurement of CT attenuation values of the main portal vein (blue), inferior vena cava (green), aorta (red), spleen (yellow), and back muscle (white), axial portal venous phase image.

### Statistical analysis

The patients were divided into two groups in a 2:1 ratio in order of the date of cancer diagnosis. Pearson’s correlation was used to assess the relationship between the difference in tumor attenuation values measured on two CT scans of the same patient and the difference in attenuation values of other organs (aorta, main portal vein, IVC, spleen, and back muscle) in the first group. The Kruskal-Wallis test was used to assess whether the differences in tumor attenuation values varied according to the tube voltage of CT scans taken at outside institutions. Based on the results of the Pearson correlation analysis, a formula was developed to correct the CT attenuation value of the tumors using univariate linear regression analysis. The performance of the developed formula was then evaluated by applying it to the second group and comparing the calculated tumor attenuation values with the actual measured values. Interobserver agreement for measuring CT attenuation values was assessed by intraclass correlation coefficient (ICC) analysis, with a two-way random-effects model and Bland-Altman analysis. ICC values were interpreted as follows: values less than 0.5, between 0.5 and 0.75, between 0.75 and 0.9, and greater than 0.90 are indicative of poor, moderate, good, and excellent reliability, respectively [9]. Results were considered statistically significant when the two-sided *P* values were less than 0.05. Statistical analyses were performed using SAS version 9.4 (SAS Institute Inc, Cary, NC, USA) and R 4.2.3 (Vienna, Austria; http://www.R-project.org).

## Results

The basic characteristics of included patients and CT scans are shown in Table 1.

**Table 1:** Basic characteristics of patients and CT scans.

	Total (n = 211)	First group (n = 141)	Second group (n = 70)
Age (years)	62 ± 12	61 ± 12	64 ± 12
Sex (male)	146 (69.2%)	91 (64.5%)	55 (78.6%)
Body mass index (kg/m^2^)	23.0 ± 3.3	22.8 ± 3.2	23.4 ± 3.5
CT interval (days)	11 ± 7	10 ± 6	13 ± 7
kVp of outside CT scans			
80	3 (1.4%)	3 (2.1%)	0 (0%)
90	5 (2.4%)	2 (1.45)	3 (4.3%)
100	65 (30.8%)	42 (29.8%)	23 (32.9%)
110	7 (3.3%)	5 (3.6%)	2 (2.9%)
120	124 (58.8%)	85 (60.3%)	39 (55.7%)
130	5 (2.4%)	4 (2.8%)	1 (1.4%)
140	2 (1.0%)	0 (0%)	2 (2.7%)

### CT attenuation values of tumor and other organs

The CT attenuation values of the tumors and other organs and the difference in CT attenuation values between the two CT scans are summarized in Table 2. The mean difference of CT attenuation values of tumors measured in two CT scans was -14.8 HU (standard deviation, 17.6; range, -80.2 - 41.0).

**Table 2:** Measured CT attenuation values of tumors and other organs.

	CT scan 1 (our institution)		CT scan 2 (outside)		Difference (CT scan 1 - scan 2)	
	Mean ± SD	Range	Mean ± SD	Range	Mean ± SD	Range
Tumor	84.1 ± 21.8	37.8 - 170.0	99.0 ± 25.6	47.4 - 213.5	-14.8 ± 17.6	-80.2 - 41.0
Aorta	161.1 ± 26.1	110.2 - 321.3	189.2 ± 38.2	121.0 - 317.5	-28.1 ± 37.6	-164.0 - 71.5
Main portal vein	164.4 ± 26.2	109.7 - 293.6	194.4 ± 38.2	119.5 - 318.5	-29.9 ± 37.8	-154.6 - 93.5
Inferior vena cava	134.8 ± 22.6	78.4 - 226.1	139.8 ± 35.2	51.6 - 254.3	-5.0 ± 31.7	-126.8 - 81.6
Spleen	124.3 ± 17.7	79.3 - 206.0	136.2 ± 22.9	86.0 - 223.0	-12.0 ± 23.2	-90.4 - 49.8
Back muscle	70.5 ± 8.7	39.8 - 100.7	66.4 ± 9.2	38.2 - 90.7	4.1 ± 9.8	-16.8 - 40.8

The measurement of CT attenuation values showed good reproducibility for the tumor and back muscle, and excellent reproducibility for the aorta, main portal vein, IVC, and spleen (Table 3). Bland-Altman plots are presented in Fig 3.

**Table 3:** Interobserber agreement for CT attenuation measurement.

	Intraclass correlation coefficient (95% confidence interval)
Tumor	0.86 (0.81 - 0.91)
Aorta	0.98 (0.98–0.99)
Main portal vein	0.96 (0.94 - 0.97)
Inferior vena cava	0.95 (0.93 - 0.97)
Spleen	0.98 (0.96 - 0.98)
Back muscle	0.76 (0.65 - 0.83)

**Fig 3:**
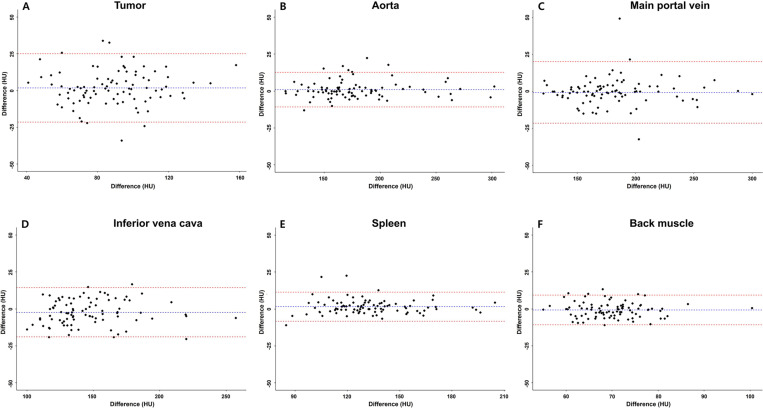
Bland-Altman plots for interobserver agreement for CT attenuation measurements.

### Formula for correcting tumor attenuation values

The Pearson correlation coefficient was the highest between the tumor and the main portal vein (*r* = 0.86, *P* <.01), followed by the tumor and the spleen (*r* = 0.73, *P* <.01), the tumor and the aorta (*r* = 0.61, *P* <.01), the tumor and the inferior vena cava (*r* = 0.45, *P* <.01), and the tumor and the back muscle (*r* = 0.37, *P* <.01) (Fig 4).

**Fig 4:**
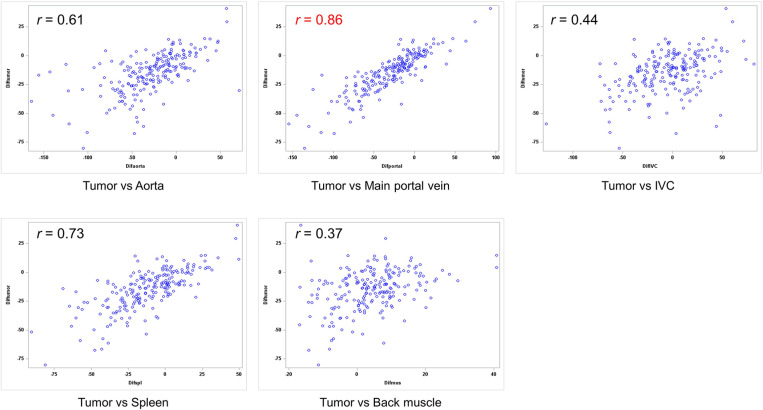
Correlation between difference of tumor attenuation and difference of various organs. The Pearson correlation coefficient is the highest between the tumor and the main portal vein (*r* = 0.86).

The formula to correct the tumor attenuation values using the main portal vein attenuation with the highest correlation was as follows:


$$CalculatedtumorattenuationvalueonCTscan2=tumorattenuationvalueonCTscan1-\left(-3.5+0.4x\left(MPVattenuationvalueonCTscan1-MPVattenuationvalueonCTscan2\right)\right)$$

There were differences in the difference in tumor attenuation values according to the tube voltage of CT scans obtained at outside institutions (*P* <.01) (Fig 5). Based on these results, two formulas were developed to calculate tumor attenuation values in patient groups with CT scans taken at 100 kVp (n = 42) and 120 kVp (n = 85), respectively at outside institutions: Calculated tumor attenuation value on CT scan 2 = tumor attenuation value on CT scan 1 - (-6.4 + 0.3 x (MPV attenuation value on CT scan 1 - MPV attenuation value on CT scan 2)) in the patient group with CT scans taken at 100 kVp, Calculated tumor attenuation value on CT scan 2 = tumor attenuation value on CT scan 1 - (-2.6 + 0.4 x (MPV attenuation value on CT scan 1 - MPV attenuation value on CT scan 2)) in the patient group with CT scans taken at 120 kVp.

**Fig 5:**
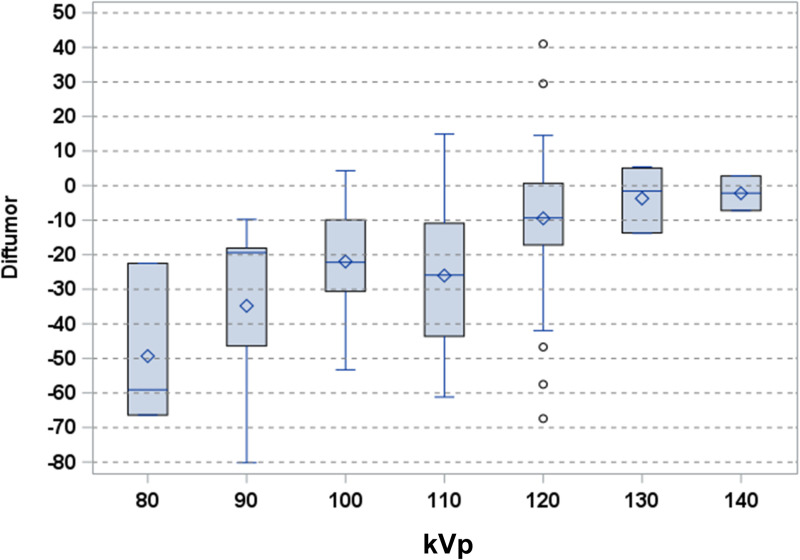
Difference in tumor attenuation values depending on tube voltage of outside CT scans.

### Validation of the developed formula

When applied to the second group, the formula developed using all patients in the first group showed a mean difference of 1.6 HU (standard deviation, 8.7; range -22.5–24.72) between calculated and actual tumor attenuation values, with a Pearson correlation coefficient of 0.95 (*P* <.01) (Fig 6A). The formula developed using patients with CT scans taken at 120 kVp at outside institutions showed a mean difference of 1.4 HU (standard deviation, 9.3; range -17.5–23.82) between calculated and actual tumor attenuation values, with a Pearson correlation coefficient of 0.91 (*P* <.01) (Fig 6B). The formula developed using patients with CT scans taken at 100 kVp at outside institutions showed a mean difference of 0.6 HU (standard deviation, 6.7; range -12.7–10.86) between calculated and actual tumor attenuation values, with a Pearson correlation coefficient of 0.97 (*P* <.01) (Fig 6C).

**Fig 6:**
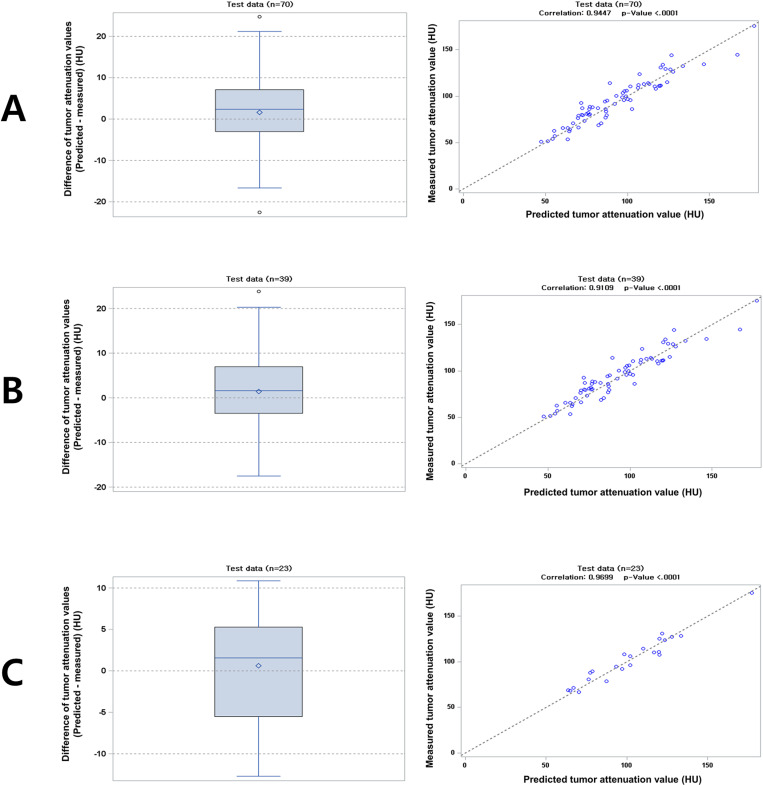
Comparison between calculated tumor attenuation values using the developed formula and measured tumor attenuation values. A.All testing group (n=70). B.Testing group with 120 kVp (n=39). C.Testing group with 100 kVp (n=23).

## Discussion

This study analyzed the correlation between the attenuation values of the tumor and various anatomical structures on two repeat CT scans in patients with advanced gastric cancer. We demonstrated that it is possible to correct for variations in tumor attenuation values due to differences in CT imaging conditions by using the attenuation value of the main portal vein. These results may be useful for characterizing tumors or evaluating their treatment response by predicting the degree of contrast enhancement based on the intrinsic characteristics of the tumor.

We measured the attenuation values of tumors and various organs on two CT scans obtained before gastric cancer treatment. Despite the relatively short interval between the two CT scans and no intervening treatment, the attenuation values measured from the two CT scans showed significant variation (mean difference, -14.8 HU; standard deviation, 17.6 HU; range, -80.2 HU - 41.0 HU), even within the same patient. This underscores the importance of caution when comparing the degree of tumor enhancement or evaluating changes in tumor enhancement based on measured attenuation values without correcting for variations in CT imaging conditions.

In this study, we assessed the correlation between the attenuation values of various organs and gastric cancers. The results showed that the attenuation value of the main portal vein exhibited the highest correlation with the tumor attenuation value. This finding aligns with a previous study investigating the correlation between vascular structures and tumor attenuation values in liver metastases of colon cancer, which also reported a correlation between the portal vein and tumor attenuation [8]. The portal venous phase is the basic imaging phase for determining the tumor stage, including distant metastasis [10], and the enhancement of gastric cancer peaks most often during this phase [11,12]. Therefore, we measured the attenuation values of gastric cancer and other organs only in the portal venous phase, characterized by full portal vein enhancement. This may explain the high correlation between the attenuation of the main portal vein and that of the tumor. Additionally, the high correlation could be attributed to the fact that the major veins of the stomach drain into the portal vein either directly or through the splenic and superior mesenteric veins [13].

At our institution, CT scans were acquired at a constant tube voltage of 120 kVp, whereas CT scans from outside institutions were acquired at various tube voltages. Using low voltages such as 100 kVp and 80 kVp results in higher CT attenuation than at 120 kVp and also increases the rate of increase in CT attenuation values with increasing iodine concentration [6]. This is well reflected in the results of our study. When considering all patients, regardless of tube voltage, the difference in tumor attenuation values increased at a ratio of 0.4 times the difference in the main portal vein attenuation values. However, when targeting only patients with CT scans obtained at 100 kVp from outside institutions, the ratio decreased to 0.3.

Our research findings could be clinically useful. For instance, when comparing the attenuation of tumors before treatment, a specific main portal vein attenuation value (e.g., the mean attenuation value of the main portal vein on CT scans taken at our institution, which was 164 HU) can be established. The adjusted tumor attenuation value can be calculated by utilizing the attenuation values of the main portal vein and the tumors measured in each patient. Similarly, when evaluating the changes in tumor attenuation values after treatment, the adjusted tumor attenuation can be calculated using the same approach. The adjusted tumor attenuation value can be used to more reliably classify the tumor phenotype or evaluate treatment response to drugs such as anti-angiogenic agents.

Our study had several limitations. First, we only measured attenuation value from a single cross-section of the tumor. There is a possibility of differences in attenuation values depending on the selected region when considering the tumor heterogeneity. Although the interobserver agreement in attenuation value measurement was good, further research may be needed to evaluate the advantages and disadvantages of measuring attenuation values by segmenting the entire tumor. Second, our study confirmed that predictive models may vary depending on differences in tube voltage. In addition to tube voltage, several other factors in the imaging protocol may influence CT attenuation values. However, due to the lack of specific imaging protocol details from outside institutions in this study, we were unable to assess the impact of these factors. Further research is needed to address this issue. Third, the histological type and degree of differentiation of gastric cancer was not considered separately. Histological factors influence the CT attenuation values and enhancement patterns of gastric cancer [14,15]. However, because most of our patients with advanced gastric cancer did not undergo surgery and received chemotherapy only after endoscopic biopsy, there were limitations in confirming the exact histological types. Therefore, additional studies considering histological factors are required to obtain more accurate predictions. Finally, our institution does not include unenhanced CT in the staging CT protocol for gastric cancer. Therefore, we only measured the attenuation value of the tumors on the portal venous phase images. Attenuation on unenhanced CT can reflect the intrinsic characteristics of a tumor. For instance, mucinous adenocarcinoma typically exhibits low attenuation on unenhanced CT. In contrast, the degree of contrast enhancement is indicative of tumor vascularity. However, tumor attenuation measured in the portal venous phase is influenced by both the unenhanced attenuation and the extent of contrast enhancement. Therefore, measuring tumor attenuation in the portal venous phase alone does not allow for the distinction between these two factors. Therefore, research targeting patient cohorts, including those with unenhanced CT scans is required to develop models that can predict the degree of contrast enhancement more accurately.

In conclusion, utilizing the attenuation value of the main portal vein allowed the correction of variations in tumor attenuation values caused by different CT imaging conditions, enabling the prediction of reproducible tumor attenuation in patients with advanced gastric cancer.

## Supporting information

S1 Data Data set to consist of the data required to replicate all study findings reported in the article. (XLSX)
